# Reevaluation of the odd chrysidid genus *Atoposega* Krombein (Hymenoptera, Chrysididae, Amiseginae)

**DOI:** 10.3897/zookeys.409.7414

**Published:** 2014-05-14

**Authors:** Lynn S. Kimsey

**Affiliations:** 1 Department of Entomology, University of California, Davis

**Keywords:** *Mahinda*, *Perissosega*, Chiang Mai, Thailand

## Abstract

The south Asian amisegine genus *Atoposega* Krombein, 1957, is reevaluated. Three new species, *A. rufithorax*, *A. striata* and *A. thailandica* are described from Thailand and the previously described species, *A. lineata* (Krombein, 1957) from Borneo, *A. rieki* (Krombein, 1957) from Myanmar and *A. simulans* Kimsey, 1986 from Malaysia are redescribed. The species, *A. decorata* Kimsey, 1995, was found to lack the generic characters diagnostic for *Atoposega*. *Atoposega* is only known from females.

## Introduction

Female *Atoposega* Krombein, 1957 are among the more striking members of the chrysidid subfamily Amiseginae. They are brightly colored and elaborately sculptured, with long, spine-like propodeal teeth (a feature shared with female *Mahinda* Krombein, 1983) and often banded wings. However, *Mahinda* females are strongly brachypterous. Thus far *Atoposega* is only known from females. The only other genus with the same Rs vein configuration is *Perissosega* Krombein, 1983 from Sri Lanka, which is known from both sexes. *Perissosega* females are fully winged and have a well-developed transverse frontal carina. No male amisegines are known that have the distinctively angulate Rs vein seen in male and female *Perissosega* and in female *Atoposega*.

**Figure 1.**
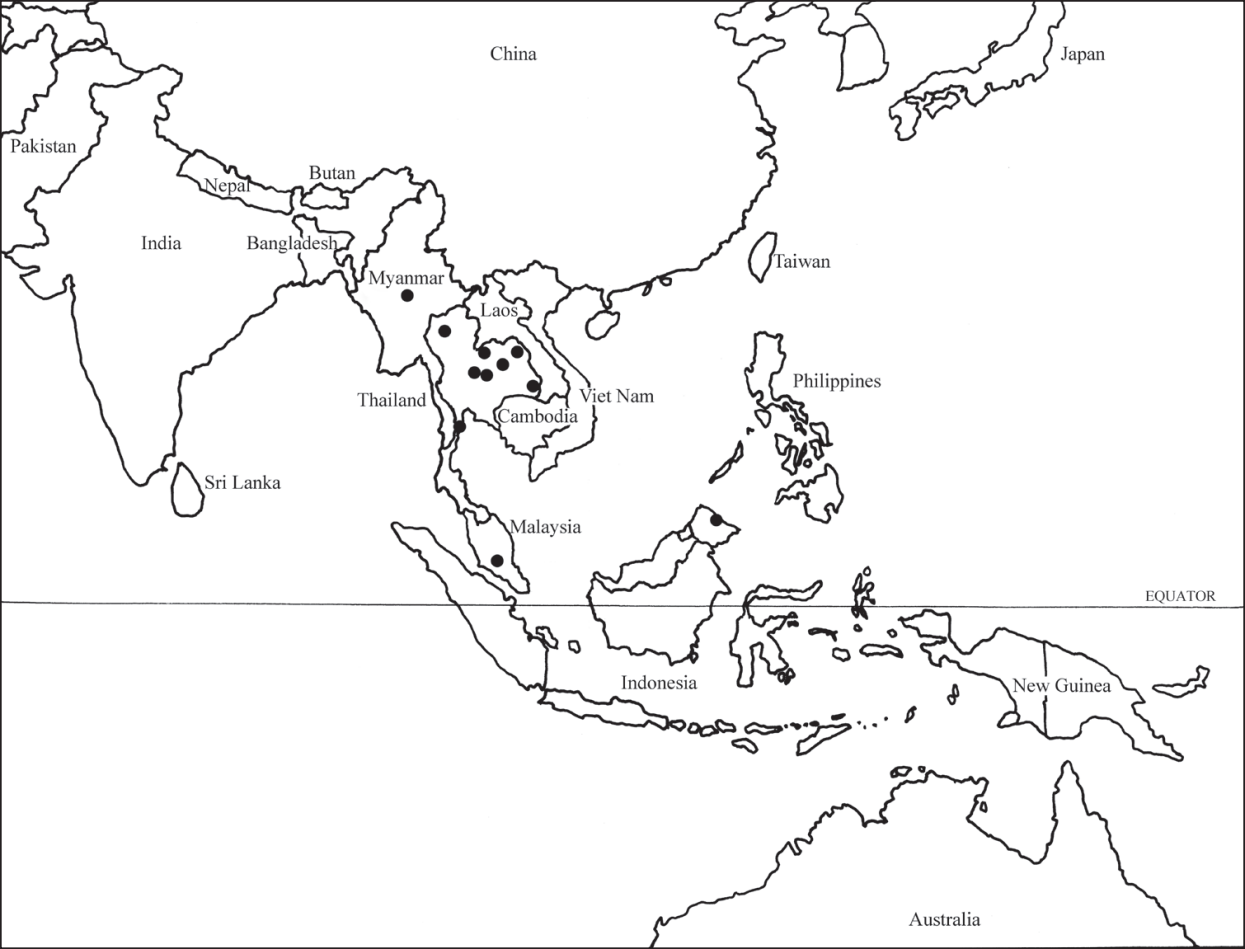
Distribution map of *Atoposega* species. The location of *Atoposega rieki* is centrally placed in-country as the actual locality is unknown.

Species of *Atoposega* are known from southern Asia (Fig. 1). The intensive survey of Hymenoptera of Thailand, a U.S. National Science Foundation project, NSF No. 0542864, headed by Michael Sharkey revealed several additional species of *Atoposega*. The one species outside of this region, *Atoposega decorata* Kimsey, 1995, from New Caledonia lacks many of the generic traits that characterize *Atoposega* and is probably not congeneric. Thus this species is not included in this study, and will be treated in a separate paper.

Nothing is known of the biology of *Atoposega* species, although it is assumed that they are parasites of walking stick eggs like other members of the subfamily.

## Materials and methods

Specimens were studied from the following institutions and/or these are the type repositories: AEI – American Entomological Institute, Gainesville, Florida, USA; BME – Bohart Museum of Entomology, University of California, Davis, USA; MCZ – Museum of Comparative Zoology, Harvard University, Cambridge, Massachusetts, USA; MNHN
– Museúm National d’Histoire Naturelle, Paris, France; QSBG – Queen Sirikit Botanic Garden, Chiang Mai, Thailand; USNM – U. S. National Museum of Natural History, Washington, D. C., USA.

Terminology used below follows that of Kimsey and Bohart (1991). Proportions of the flagellomeres are based on the greatest length versus the broadest part of the article, generally the apical margin.

## Taxonomy

### Genus Atoposega

Krombein

http://species-id.net/wiki/Atoposega

Atopogyne Krombein, 1957: 184. Type: *Atopogyne lineata* Krombein, 1957: 186. Nec Forel 1911. Original designation
Atoposega Krombein, 1960: 33. Replacement name for *Atopogyne* Krombein.

#### Diagnosis.

Female *Atoposega* most closely resemble those of *Mahinda* Krombein based on the acute or spine-like propodeal angles and mesopleuron with-well developed omaulus. Female *Atoposega* differ from *Mahinda* as they are fully winged (all known *Mahinda* are strongly brachypterous), the hindcoxa has with two longitudinal carina (one or none in *Mahinda*), and the mesopleuron has a dorsally carinate and U-shaped posteromedial groove (a narrow, parallel-sided longitudinal groove in *Mahinda*). In addition *Mahinda* females have two sharp submedial angles above the posterior propodeal declivity, which do not occur in *Atoposega*. *Perissosega* females are fully winged, but unlike *Atoposega* and *Mahinda* have a transverse frontal carina, and lack an omaulus. *Atoposega* can be distinguished from other amisegine genera by these characters, and by the dentate tarsal claws, malar space with a vertical sulcus, frons without transverse carina, vertex without longitudinal welt, pronotum with posteromedial longitudinal pit, and short sulcus and pit adjacent to lateral posterior lobe, mesopleuron without scrobal sulcus, metanotal dorsal enclosure usually V-shaped, propodeum with two dorsomedial decumbent angles and posterior declivity smooth and impunctate, with longitudinal medial carina, and forewing with an arcuate Rs vein.

#### Generic description.

Head: occipital carina well-developed, visible laterally; eye with tiny sparse setulae, encircled by carina; scapal basin deep, wide and coarsely cross-ridged; malar space with vertical groove; female flagellum short, fusiform and flattened on one surface. Mesosoma: pronotum with posteromedial groove and deep pit before lateral lobe, 0.8–0.9× as long as combined lengths of scutum, scutellum and metanotum; scutum with notauli deep and narrow, without parapsides; mesopleuron evenly punctate, omaulus well-developed, scrobal sulcus absent, posteromedial fossa U-shaped, carina edged; metanotum elongate, subequal in length to scutellum, with triangular medial enclosure; propodeum dorsal surface bending abruptly to posterior declivity, lateral angles long, spike-like; hindcoxa with two longitudinal, dorsobasal carinae; tarsal claw with large medial tooth; female fully winged; forewing R1 clearly indicated, medial vein arising before cu-a, Rs extended at abrupt angle by dark streak; wings densely setose, often banded. Metasoma: sternum I produced into large basal keel.

#### Distribution.

*Atoposega* species have been collected in Myanmar, Borneo, Thailand and Malaysia.

#### Key to the species of *Atoposega*

**Table:** 

1	Metasomal tergum II with dense fine longitudinal scratches laterally, extending length of tergum, joining posteriorly and appearing U-shaped (as in Fig. 7)	2
–	Metasomal tergum II with apicolateral patch of dense fine longitudinal scratches, only extending part of tergal length, not joining posteriorly or appearing U-shaped	4
2	Midocellus 1.0-1.5 midocellar diameters from nearest eye margin; wing banded, with contrasting bands of dark brown alternating with untinted or whitish bands; Myanmar	*Atoposega rieki* Krombein
–	Midocellus 2 or more midocellar diameters from nearest eye margin; wing unbanded, brown-tinted	3
3	Metanotum punctate without medial ridge; flagellomere II dark brown; Thailand	*Atoposega rufithorax* sp. n.
–	Metanotum areolate with medial ridge; flagellomere II partly to entirely whitish; Thailand	*Atoposega striata* sp. n.
4	Flagellomere I 4× as long as broad; hindocellus less than one diameter from nearest eye margin in dorsal view; forewing with contrasting bands of dark brown alternating with untinted or whitish bands; Thailand	*Atoposega thailandica* sp. n.
–	Flagellomere I 3× as long as broad; hindocellus more than one diameter from nearest eye margin in dorsal view; forewing banded or not	5
5	Wing with contrasting bands of dark brown alternating with untinted or whitish bands (Fig. 8); metasomal tergum I without lateral patch of fine, dense longitudinal scratches; propodeal posterior surface cross-ridged; Borneo	*Atoposega ineata* Krombein
–	Wing without contrasting bands of dark and light, brown-tinted; metasomal tergum I with lateral patch of fine, dense longitudinal scratches; propodeal posterior surface cross-ridged; Malaysia	*Atoposega simulans* Kimsey

### Atoposega lineata

(Krombein)

http://species-id.net/wiki/Atoposega_lineata

Figures 2 , 8

Atopogyne lineata Krombein, 1957: 186. Holotype female; Borneo: Sandakan (USNM).

#### Material examined.

Malaysian Borneo: Sandakan. Only the type series has been seen.

#### Diagnosis.

This species most closely resembles *Atoposega simulans* and less so *Atoposega thailandica*. All lack the dense U-shaped scratches on metasomal tergum II. *Atoposega lineata* and *Atoposega thailandica* both have banded wings, but *Atoposega lineata* can be distinguished by the hindocellus separated from the eye margin by more than one diameter and flagellomere I less than 4× as long as broad.

#### Female description.

**Figures 2–6.**
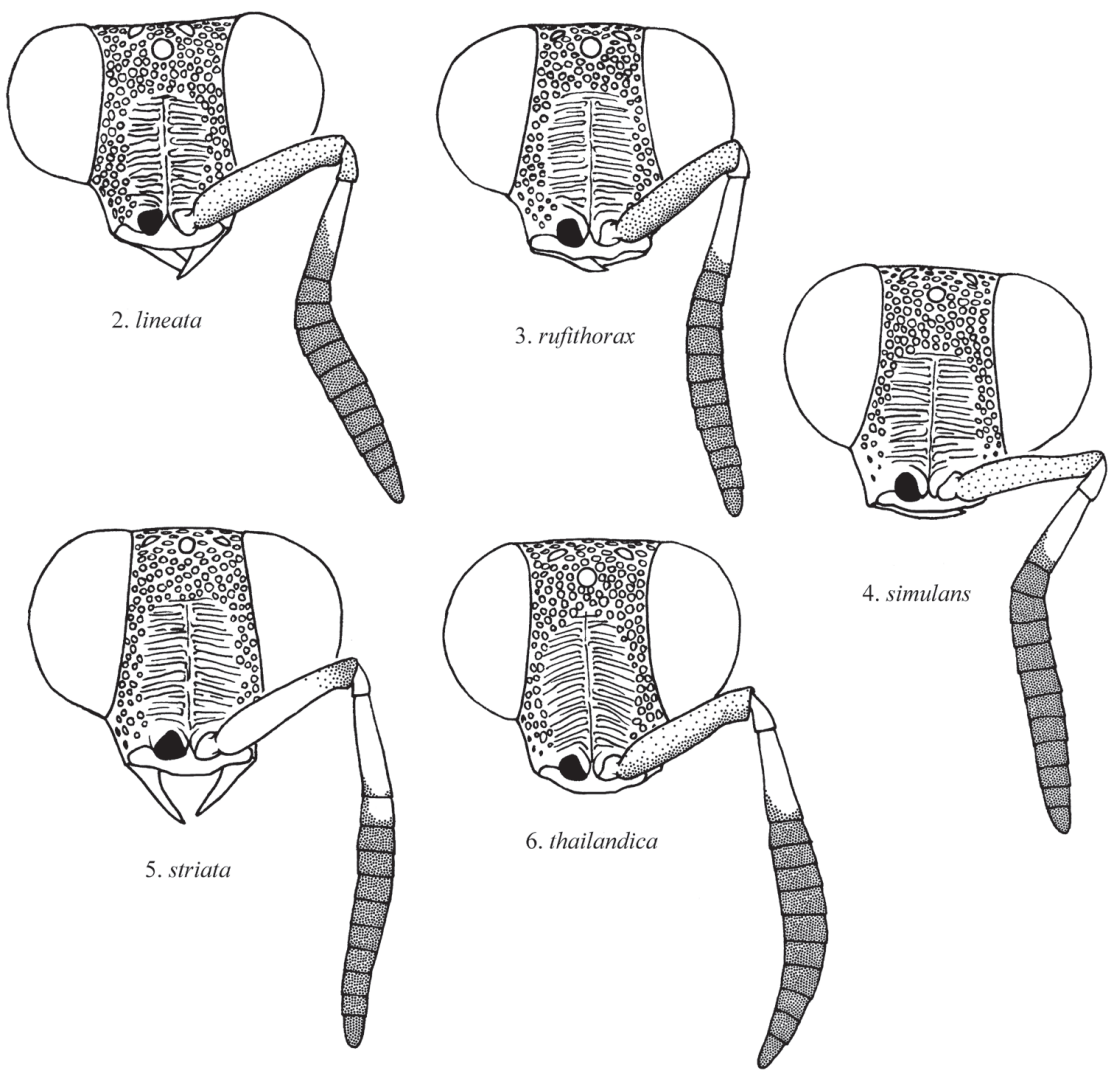
Front view of female *Atoposega* face, with one antenna removed.

Body (Fig. 8): length 6 mm. Head: face (Fig. 2); scapal basin cross-ridged medially; frons with punctures contiguous; malar space 3 midocellus diameters long; head 0.8× as long as wide; midocellus 2.6–2.7 midocellus diameters from ocular margin in front view; ocelli arranged in triangle broader between hindocelli than between hind and midocelli; hindocellus separated from ocular margin by 1.5 ocellar diameters in dorsal view; clypeus somewhat convex apically; subantennal distance 0.8 midocellus diameters: flagellomere I length 3× breadth; flagellomeres II and III 0.7× as long as broad; flagellomere XI 1.2× as long as broad. Mesosoma: pronotal, scutal and scutellar punctures large, contiguous, separated by longitudinal ridges; pronotum and scutum subequal in length; scutum with notauli well-developed anteriorly; mesopleural punctures contiguous; metanotum 1.3× as long as scutellum, medial enclosure coarsely areolate, with medial ridge; propodeal dorsal surface coarsely areolate, posterior surface with medial longitudinal carina, enclosure smooth, impunctate on either side; forefemur with tiny contiguous punctures dorsally, punctures becoming separated by 1 puncture diameter ventrally; hindfemur with tiny contiguous punctures dorsally, punctures becoming larger and contiguous ventrally, with longitudinal impunctate band ventrally; metapleuron and propodeal side coarsely cross-ridged. Metasoma: tergum I polished, impunctate; tergum II polished, impunctate medially from anterior to posterior margins, laterally with dense, tiny punctures and fine, dense longitudinal scratches; terga III-IV polished with scattered tiny punctures; sternum II with punctures tiny, 5–10 puncture diameters apart, III-IV punctures tiny, contiguous to 0.5 puncture diameters apart. Color: head black; mesosoma red; propodeum black dorsally and posteriorly, laterally and lateral tooth partly to completely red; wings banded, dark brown-tinted, with un-tinted band across wing at Rs and near base; antenna with scape, pedicel red, flagellomere I paler red dorsally, browner ventrally, flagellomeres II-IX dark brown; legs red, midcoxae and hindfemora may have brown lateral spot; metasoma black with faint metallic green tints.

**Figures 7–11.**
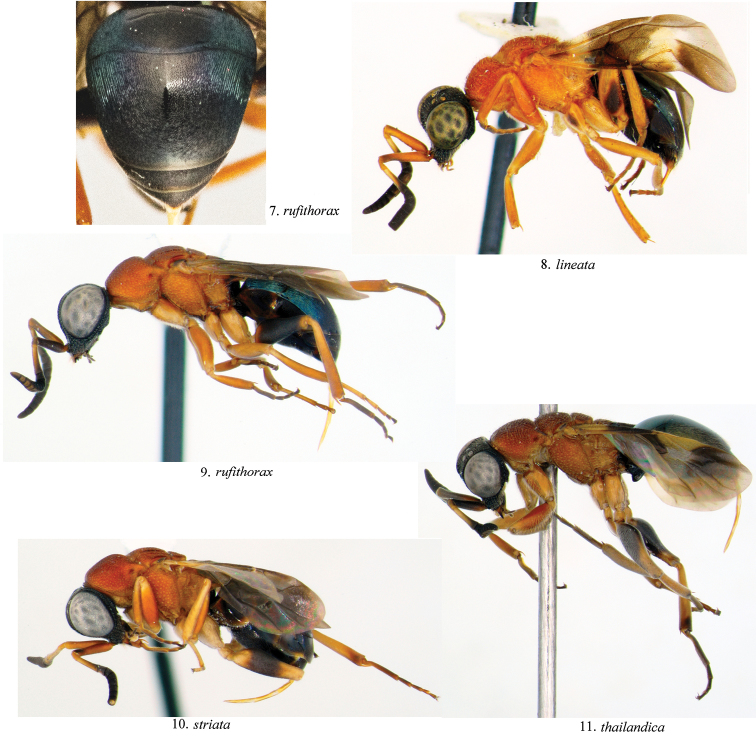
Lateral view of female *Atoposega*.

### Atoposega rieki

(Krombein)

http://species-id.net/wiki/Atoposega_rieki

Atopogyne rieki Krombein 1957: 187. Holotype female; “Birmanie” (Myanmar) (PARIS).

#### Material examined.

Type unavailable for study; Myanmar.

#### Diagnosis.

*Atoposega rieki* most closely resembles *Atoposega rufithorax* and *Atoposega striata* based on the presence of fine dense longitudinal scratches on metasomal tergum II that join posteriorly, appearing broadly U-shaped. It can be distinguished from those species by the narrower subantennal distance, midocellus separated from the eye margin by less than two midocellar diameters, and banded wings.

#### Female description

(based on Krombein 1957). Body: length 6 mm. Head: face scapal basin transversely ridged medially; frons with punctures deep, contiguous; malar space 3.5 midocellus diameters long; head 0.9× as long as wide; midocellus 1.3 midocellus diameters from ocular margin; ocelli arranged in isosceles triangle; hindocellus separated from ocular margin by 0.9 diameter; clypeus apicomedially indented; subantennal distance 0.7 midocellus diameters: flagellomere I length 3.7× breadth; flagellomere II as long as broad. Mesosoma: pronotal, scutal and scutellar punctures 0.3–0.5 puncture diameter apart; pronotum 0.6× as long as scutum, scutellum and metanotum combined; scutum with notauli well-developed anteriorly, broader posteriorly; mesopleuron with punctures contiguous to 0.5 puncture diameter apart; metanotum 0.9× as long as scutellum; hindfemur minutely, finely rugulose ventrally. Metasoma: tergum I smooth, impunctate in medial half, lateral fourth finely, longitudinally carinate; tergum II with basal triangular, finely punctate area, laterally with dense, longitudinal carinae joined posteriorly into U-shape; terga III and IV finely, densely punctate; sterna II and III with large, dense punctures. Color: head black; antenna dark brown, except scape, pedicel and flagellomere I paler basally and apex of flagellomere I blackish; flagellomeres II-XI blackish; thorax red, except dorsal and posterior face of propodeum black; legs brown, except coxae, trochanters, femora and ibiae narrowly basally red, hindtibial apex and venter of hindfemur dark brown; metasoma shiny black, with faint green tints on terga I-II; wing membrane with alternating pale or brown bands.

### Atoposega rufithorax

Kimsey sp. n.

http://zoobank.org/56C23D93-6921-4396-9EAA-DB97766835C3

http://species-id.net/wiki/Atoposega_rufithorax

Figures 3 , 7 , 9

#### Type material.

Holotype female: Thailand: Chaiyaphum Prov., Tat Tone NP, Phu hang sing, 15°58.723'N, 102°2.231'E, 290m, Malaise trap, 19–26/vii/2006, T. Jaruphan & O. Budsawong, T226 (QSBG). Paratypes: 9 females: 1 female: dry dipterocarp forest, 15°59.037'N, 102°2.103'E, 250m, Malaise trap, 25–27/vi/2006, M. Sharkey, T10; 1 female: Khonkaen Prov., Nam Pong NP, 16°37.377'N, 102°34.454'E, Malaise Trap, 5–12/vii/2006, K, Jaidee, T106; 1 female: 12–19/vii/2006, K. Jaidee, T110; 1 female: 16°37.201'N, 102°34.481'E, Malaise trap, 5–12/vii/2006, K. Jaidee, T107; 1 female: 12–19.vii.2006, K. Jaidee, T112; 1 female: Prachuab Khiri Khan Prov., Khao Sam Roi Yot NP, Laem Sala beach, 12°12.234'N, 100°0.767'E, Malaise trap, 10–17/viii/2008, Yai & Amnad, T3010; 1 female: Phetchabun Prov., Nam Nao NP, hill evergreen forest, 16°44.387'N, 101°34.531'E, 838m, pan trap, 28–29/v/2007, N. Hongyothi & L. Janteab, T2424; 1 female: helicopter landing ground, 16°43.113'N, 101°35.134'E, 889 m, Malaise trap, 10–17/vii/2006, N. Hongyothi, T273; 1 female: Khao Kho NP office, 16°39.55'N, 101°8.123'E, 230m, Malaise trap, 12–19/vii/2006, S. Chatchumnan & S. Singtong, T167 (QSBG, BME).

#### Diagnosis.

This is one of three species, including *Atoposega striata* and *Atoposega rieki*, with fine dense longitudinal carinae on the sides of metanotal tergum II that join posteromedially. It can be distinguished from those species by the lack of an elevated, medial metanotal ridge, and the scutellum roughly planar with the metanotum and separated from the metanotum by a deep notch in side view.

#### Female description.

Body (Fig. 9): length 3.5–4.5 mm. Head: face (Fig. 3); scapal basin finely, cross-ridged medially without discrete, longitudinal medial ridge; frons with punctures contiguous; malar space 3.5 midocellus diameters long; head 0.8× as long as broad in front view; midocellus 2.0–2.3 midocellus diameters from ocular margin; hindocelli further apart than midocellar-hindocellar distance; hindocellus separated from ocular margin by 0.7 hindocellar diameter; clypeus slightly concave apicomedially, punctures small, irregular; subantennal distance 0.8–0.9 midocellus diameters: flagellomere I length 5× breadth; flagellomere II 0.9× as long as broad; flagellomere III 0.7× as long as broad; flagellomere XI 1.5× as long as broad. Mesosoma: pronotal, scutal and scutellar punctures 0.5–1.0 puncture diameter apart, finely, densely striate between punctures; pronotum and scutum subequal in length; scutal notauli deep for entire length of scutum; mesopleuron without depression from pronotal lobe to scrobe, punctures large, deep, contiguous; metanotum 0.7× as long as scutellum, medial enclosure with large irregular punctures, without medial ridge; scutellum and metanotum subplanar in lateral view; propodeal dorsal surface coarsely areolate, posterior surface with carina margined medial enclosure, with longitudinal medial ridge; metapleuron and propodeal side with fine dense cross-ridging; forefemur with contiguous, tiny punctures dorsally and ventrally; hindfemur with dense tiny punctures dorsally, ventrally punctures becoming larger and reticulate. Metasoma: tergum I medial half impunctate, lateral fourth densely longitudinally striate, tergum II medially with impunctate band extending half length of tergum, becoming densely finely punctate extending laterally and becoming finely longitudinally striate, striae joining posteriorly becoming U-shaped (Fig. 7); terga III and IV with dense tiny, contiguous striatiform punctures; sterna II-IV with small punctures separated by 0.5–1.0 puncture diameters. Color: head black; scape bicolored, red to light brown ventrally, blackish dorsally; pedicel and flagellomere I pale red to brown; flagellum blackish; thorax and legs red, becoming paler ventrally, including all or most of lateral propodeum and propodeal teeth, propodeum black dorsally and posteriorly, hindtibia blackish to dark brown, with pale joints; metasomal segments black, terga with metallic bluish green tints, sternum I may be brownish; wing membrane brown-tinted, unbanded.

#### Etymology.

The name refers to the red coloration of the thorax.

### Atoposega simulans

Kimsey

http://species-id.net/wiki/Atoposega_simulans

Figure 4

Atoposega simulans Kimsey 1986: 153. Holotype female; Malaysia: Pasoh Forest Res., Negri Sembilan (AEI).

#### Material studied.

Malaysia: Negri Sembilan; Thailand: Loei Prov., Phu Ruea NP; Prachuap Khiri Khan Prov., Khan Sam Rui Yot NP; Chaiyaphum Prov., Tai Tome NP, Pha Bin Ngan NP; Ubon Ratchathani, Pha Taem NP; Khon Kaen Prov., Nam Pong NP; Phetchabun Prov., Khao Kho NP, Nam Nao NP; Sakon Nakhon Prov., Phu Pham NP; in the months of December, January and April-October; 22 females were examined including the holotype (BME, AEI).

#### Diagnosis.

This species most closely resembles *Atoposega lineata* based on dimensions of the flagellomere I, the lack of longitudinal striae on metasomal tergum II and the hindocellus separated from the nearest eye margin by more than one diameter. *Atoposega simulans* can be distinguished from *Atoposega lineata* by the lack of lateral longitudinal scratches on metasomal tergum I and evenly brown-tinted wings.

#### Female description.

Body: length 2.5–5.0 mm. Head: face (Fig. 4); scapal basin with dense, coarse cross-ridges medially; frons with punctures large, contiguous; malar space 3.0–3.5 midocellus diameters long; head width 1.2–1.3× length; midocellus 2.5 midocellus diameters from ocular margin; ocelli arranged in broad triangle; hindocellus separated from ocular margin by 1.2 diameters in dorsal view; clypeus flattened apically; subantennal distance 1 midocellus diameter long; flagellomere I length 3× breadth; flagellomere II 0.6× as long as broad. Mesosoma: pronotal, scutal and scutellar punctures large, coarse and contiguous; pronotum half as long as combined lengths of scutum, scutellum and metanotum; scutum with notauli deeper posteriorly than anteriorly; mesopleuron with large, deep, contiguous punctures; metanotum 0.6–0.8× as long as scutellum, medial enclosure areolate, with strong medial, longitudinal ridge; propodeal dorsal surface coarsely areolate, posterior surface with medial enclosure with medial, longitudinal ridge and coarse cross-ridging laterally; metapleuron and propodeal side with coarse cross-ridging; forefemur with tiny, contiguous punctures dorsally, ventrally punctures 2–3 puncture diameters apart; hindfemur with tiny, contiguous punctures dorsally, ventrally punctures separated by 0.5–1.0 puncture diameters, with broad, longitudinal impunctate, polished band medially; metapleuron and propodeal side coarsely cross-ridged. Metasoma: terga I and II polished and impunctate medially, lateral third with fine longitudinal scratches and punctures, punctures 1 puncture diameter apart, scratches merging posteriorly forming U-shape; terga III-IV with dense, contiguous tiny punctures; sternum II with tiny, widely separated punctures 5–10 puncture diameters apart; sterna III-IV punctures tiny, dense and contiguous to 0.5 puncture diameters apart. Color: head black; scape light brown; pedicel and flagellomere I whitish, except apex of flagellomere I blackish; flagellomeres II-XI blackish; thorax red, except dorsal and posterior face of propodeum black; legs and coxae red, except foretarsomeres, hindtibal apex and venter of hindfemur dark brown; metasoma shiny black, with faint green tints on terga I-II; wing membrane brown-tinted, with dark bands across wing at medial vein and apex of Rs alternating with pale bands.

### Atoposega striata

Kimsey sp. n.

http://zoobank.org/1BF8067D-553A-4D70-B7AE-423832348296

http://species-id.net/wiki/Atoposega_striata

Figures 5 , 10

#### Type material.

Holotype female: Thailand: Chaiyaphum Prov., Tat Tone NP, Phu hang sing 15°58.723'N, 102°02.231'E, Malaise trap, 19–26/vii/2006, T. Jaruphan & O. Budsawong, T1226 (QSBG). Paratypes. 13 females: 1 female: dry dipterocarp forest, 15°59.037'N, 102°2.103'E, 250m, Malaise trap, 29/vi/2006, L. Ittichan, T30; 1 female: entrance to Pa Eang waterfall, 15°57.520'N, 101°54.442'E, 297m, Malaise trap, 12–19/x/2006, T. Jaruphan, T681; 1 female: 15°58.538'N, 102°02.153'E, 280m, pan trap, 6–7/i/2007, T. Jaruphan & O. Budsawong, T1552; 1 female: Thung Dok Kra Jeow, dipterocarp forest, 15°38.208'N, 101°23.556'E, 720m, Malaise trap, 7–13/i/2007, K. Sa-nog & B. Adnafai, T1458; 2 females: Khonkaen Prov., Nam Pong NP, 16°37.377'N, 102°34.454'E, Malaise Trap, 12–19/vii/2006, K. Jaidee, T111; 1 female: Prachuab Khiri Khan Prov., Khao Sam Roi Yot NP, Laem Sala beach, 12°12.234'N, 100°0.767'E, Malaise trap, 10–17/viii/2008, Yai & Amnad, T3010; 1 female: 17–24/viii/2008, Yai & Sorat, T3016; 1 female: 200 m s checkpoint 1, 12°12.789'N, 99°58.662'E, Malaise trap, 28/ix-5/x/2008, Y. Amnad, T4102; 1 female: Loei Prov., Phu Kradueng NP, Nampong/Pong Neep forest unit, 16°56.59'N, 101°41.61'E, 273m, Malaise trap, 19–26/iv/2008, T. Phatai, T5130; 1 female: forest protection unit 5, 16°50.66'N, 101°41.5'E, 420m, Malaise trap, 12–19/vi/2008, T. Phatai, T5048; 1 female: Ubon Ratchathani Prov., Pha Taem NP, west of Huay Pok forest unit, 15°37.321'N, 105°36.982'E, 419m, Malaise trap, 6–13/x/2006, T719; 1 female: Sakon Nakhon Prov., Phu Phan NP, behind office, 17°3.543'N, 103°58.452'E, 312m, Malaise trap, 8–14/vii/2006, W. Kongnara, T197 (QSBG, BME).

#### Diagnosis.

This species is closest to *Atoposega rufithorax* based on dimensions of the flagellomeres, length of the subantennal distance and lack of a posterior propodeal enclosure. It can be distinguished from *Atoposega rufithorax* by the presence of an elevated, medial metanotal ridge, the scutellum elevated above metanotum and separated from the metanotum by a deep notch in side view, and clypeal apex truncate not broadly rounded as in *Atoposega rufithorax*.

#### Female description.

Body (Fig. 10): length 3–5 mm. Head: face (Fig. 5); scapal basin coarsely, cross-ridged medially, with medial ridge; frons with punctures contiguous; malar space 3.5 midocellus diameters long; head 0.8× as long as wide in front view; midocellus 2.5 midocellus diameters from ocular margin; hindocelli further apart than midocellar-hindocellar distance; hindocellus separated from ocular margin by 0.8× hindocellar diameter; clypeus flat apicomedially, punctures small, irregular; subantennal distance 1.0–1.2 midocellus diameters: flagellomere I length 3.8× breadth; flagellomere II as 0.8× as long as broad; flagellomere III 0.6× as long as broad, flagellomere XI 1.6× as long as broad. Mesosoma: Pronotal, scutal and scutellar punctures 0.5–1.0 puncture diameter apart, finely, densely striate between punctures; pronotum as long as scutum in length; scutal notauli deeper posteriorly than anteriorly, extending entire length of scutum; mesopleuron without depression from pronotal lobe to scrobe, punctures large, deep, contiguous; metanotum 0.9× as long as scutellum, medial enclosure areolate, with strongly elevated medial ridge; propodeal dorsal surface coarsely areolate, posterior surface with medial enclosure with elevated marginal carina particularly well-developed dorsally, with longitudinal medial ridge and transverse ridges; metapleuron and propodeal side densely cross-ridged; forefemur and hindfemur densely punctate-reticulate ventrally. Metasoma: terga I and II polished and impunctate medially, lateral third with fine longitudinal scratches and punctures, punctures 1 puncture diameter apart, scratches merging posteriorly forming U-shape; terga III-IV with dense, contiguous tiny punctures; sterna II-IV with dense small punctures 0.5–1.0 puncture diameters apart, punctures smaller and somewhat denser on III-IV. Color: head black; scape bicolored, red to light brown ventrally, blackish dorsally; pedicel and flagellomere I pale red to brown; flagellum blackish; thorax and legs red, becoming paler ventrally, including all or most of lateral propodeum and propodeal teeth, hindtibia black, with pale joints; propodeum black dorsally and posteriorly; metasomal segments black, terga with metallic green tints, sternum I may be brownish; wing membrane brown-tinted.

#### Etymology.

The species name refers to the fine longitudinal scratches on the dorsum of the mesosoma.

### Atoposega thailandica

Kimsey sp. n.

http://zoobank.org/55B5117A-FE01-44D8-AEAD-F81381415DB5

http://species-id.net/wiki/Atoposega_thailandica

Figures 6 , 11

#### Type material.

Holotype female: Thailand: Chiang Mai Prov., Doi Chiangdao NP Water reservoir, 19°24.419'N, 98°55.237'E, 549m, Malaise trap, 11–18.ix.2007, S. Jugsu & A. Watwanich, T5686 (QSBG). Paratypes: 6 females: 1 female: Doi Inthanon NP, 700 m, 18°32N, 98°36E, Malaise trap, 8–15/vii/2006, Y. Areeluck, T62; 1 female: Phetchabun Prov., Nam Nao NP, pine forest/Sambon 1, 16°42.47'N, 101°35.26'E, 872m, Malaise trap, 5–12/iv/2007, L. Janteab, T4948; 1 female: helicopter landing ground, 16°43.113'N, 101°35.134'E 889m, Malaise trap 17–24/vii/2006, N. Hongyothi leg. T276; 1 female, Loei Prov., Phu Ruea NP, 17°27.829'N, 101°21.36'E, 691m, pan trap, 5–6/xii/2006, P. Tumtip, T1254; 1 female: Sakon Nakhon Prov., Phu Phan NP, 17°9.824'N, 103°54.511'E, 199m, Malaise trap, 25–31/x/2006, W. Kongnara, T709; 1 female: Chaiyaphum Prov., Pa Hin Ngam NP, car park at Tung Dok Grajeaw, 15°38.438'N, 101°23.576'E 780m, pan trap 7–8/vii/2006, Kratae Sa-nog & Buakaw Adnafai, T325 (QSBG, BME).

#### Diagnosis.

This species most closely resembles *Atoposega lineata* and *Atoposega simulans* based on the lack of fine dense U-shaped striae on metasomal tergum II. The banded wings resemble those of *Atoposega rieki* and *Atoposega lineata*. *Atoposega thailandica* can be distinguished from these species by the combination of flagellomere I 4× as long as broad (versus 3×) and hindocellus separated by less than 1 diameter from the nearest eye margin.

#### Female description.

Body (Fig. 11): length 5–6 mm. Head: face (Fig. 6); scapal basin cross-ridged medially; frons with punctures contiguous; malar space 2.6 midocellus diameters long; head 0.9× as long as wide; midocellus 2.2 midocellus diameters from ocular margin; hindocelli slightly further part than hindocelli and midocellus; hindocellus separated from ocular margin by 0.8 diameter; clypeus coarsely punctate and flattened apically; subantennal distance 0.9 midocellus diameters long; flagellomere I length 4× breadth; flagellomere II 0.5–0.6× as long as broad; flagellomere III 0.4× as long as broad; flagellomere XI 1.8× as long as broad. Mesosoma: pronotal, scutal and scutellar punctures large, contiguous, separated by longitudinal ridges; pronotum and scutum subequal in length; scutum with notauli deeper posteriorly than anteriorly; mesopleuron with large, contiguous punctures; metanotum 0.8× as long as scutellum, medial enclosure coarsely areolate, with medial ridge; propodeal dorsal surface coarsely areolate, posterior surface with numerous cross-ridges on either side of longitudinal medial ridge; metapleuron and propodeal side coarsely cross-ridged; hindcoxa with two dorsal, longitudinal carinae, merging basally, elevated into basal tooth or angle; forefemur ventrally with tiny, contiguous punctures; hindfemur ventrally with longitudinal polished impunctate band. Metasoma: tergum highly polished, medial two-thirds impunctate, laterally with zone of small punctures, separated by 0.5–1.0 puncture diameters; tergum II medial third polished impunctate on anterior half of tergum, lateral third punctate, with punctures separated by 0.5–1.0 puncture diameters, becoming slightly striatiform laterally; terga III-IV with dense, small, contiguous punctures; sterna II-IV with punctures separated by 0.5–1.0 puncture diameter, punctures largest on II becoming progressively smaller on subsequent sterna. Color: head black; scape bicolored, red to light brown ventrally, blackish dorsally; pedicel and flagellomere I pale red to brown; flagellum blackish; mesosoma and legs red, becoming paler ventrally, including all or most of lateral propodeum and propodeal teeth, hindtibia black with pale joints; propodeum black dorsally and posteriorly; metasomal segments black, terga with brassy tints, sternum I may be brownish; wing membrane bicolored, membrane and setae dark brown tinted, with broad pale band across wing at stigma.

#### Etymology.

The species is named after the country of collection.

## Supplementary Material

XML Treatment for Atoposega

XML Treatment for Atoposega lineata

XML Treatment for Atoposega rieki

XML Treatment for Atoposega rufithorax

XML Treatment for Atoposega simulans

XML Treatment for Atoposega striata

XML Treatment for Atoposega thailandica

## References

[B1] Forel A (1911) Fourmis de Bornéo, Singapore, Ceylan, etc. récoltées par MM. Haviland, Green, Winkler, Will, Hose, Roepke et Waldo. Revue Suisse de Zoologie 19: 23-62

[B2] Kimsey LS (1986) New species and genera of Amiseginae from Asia. Psyche 93: 153-165. doi: 10.1155/1986/31631

[B3] Kimsey LS (1995) New amisegine wasps from Southeast Asia (Hymenoptera: Chrysididae). Proceedings of the Entomological Society of Washington 97(3): 590-595

[B4] Kimsey LS Bohart RM (1991 (1990)) The Chrysidid Wasps of the World. Oxford University Press, ix + 652

[B5] Krombein KV (1957) A generic review of the Amiseginae, a group of phasmatid egg parasites, and notes on the Adelphinae. Transactions of the American Entomological Society 82: 147-215

[B6] Krombein KV (1960) Additions to the Amiseginae and Adelphinae. Transactions of the American Entomological Society 86: 27-39

[B7] Krombein KV (1983) Biosystematic studies, XI: A monograph of the Amiseginae and Loboscelidiinae. Smithson. Contrib. Zool. (376): 1–79. doi: 10.5479/si.00810282.376

